# WASp-dependent actin cytoskeleton stability at the dendritic cell immunological synapse is required for extensive, functional T cell contacts

**DOI:** 10.1189/jlb.2A0215-050RR

**Published:** 2015-11-20

**Authors:** Dessislava Malinova, Marco Fritzsche, Carla R. Nowosad, Hannah Armer, Peter M. G. Munro, Michael P. Blundell, Guillaume Charras, Pavel Tolar, Gerben Bouma, Adrian J. Thrasher

**Affiliations:** * Molecular Immunology Unit, University College London Institute of Child Health, London, United Kingdom;; † London Centre for Nanotechnology and Department of Cell and Developmental Biology, University College London, London, United Kingdom;; ‡ Division of Immune Cell Biology, Medical Research Council National Institute for Medical Research, The Ridgeway, Mill Hill, London, United Kingdom;; § Imaging Unit, University College London Institute of Ophthalmology, London, United Kingdom; and; ¶ Great Ormond Street Hospital for Children, National Health Service Foundation Trust, London, United Kingdom

**Keywords:** DC, ICAM-1, podosomes, FRAP, Arp2/3

## Abstract

The immunological synapse is a highly structured and molecularly dynamic interface between communicating immune cells. Although the immunological synapse promotes T cell activation by dendritic cells, the specific organization of the immunological synapse on the dendritic cell side in response to T cell engagement is largely unknown. In this study, confocal and electron microscopy techniques were used to investigate the role of dendritic cell actin regulation in immunological synapse formation, stabilization, and function. In the dendritic cell-restricted absence of the Wiskott-Aldrich syndrome protein, an important regulator of the actin cytoskeleton in hematopoietic cells, the immunological synapse contact with T cells occupied a significantly reduced surface area. At a molecular level, the actin network localized to the immunological synapse exhibited reduced stability, in particular, of the actin-related protein-2/3-dependent, short-filament network. This was associated with decreased polarization of dendritic cell-associated ICAM-1 and MHC class II, which was partially dependent on Wiskott-Aldrich syndrome protein phosphorylation. With the use of supported planar lipid bilayers incorporating anti-ICAM-1 and anti-MHC class II antibodies, the dendritic cell actin cytoskeleton organized into recognizable synaptic structures but interestingly, formed Wiskott-Aldrich syndrome protein-dependent podosomes within this area. These findings demonstrate that intrinsic dendritic cell cytoskeletal remodeling is a key regulatory component of normal immunological synapse formation, likely through consolidation of adhesive interaction and modulation of immunological synapse stability.

## Abstract (precis)

Novel DC podosomes surround the central MHCII cluster to stabilize the IS; a driver role for the DC actin cytoskeleton.

## Introduction

A crucial step for successful T cell activation is conjugated interaction at the IS, an organized contact interface thought to allow optimal antigen recognition and signal transduction. IS formation requires the dynamic remodeling of the actin cytoskeleton to distribute membrane areas spatially with distinct protein compositions. Our knowledge of the IS and its role in T cell activation has improved greatly over the last couple of decades (reviewed in ref. [1]). Most studies have focused on the T cell, showing the importance of the T cell actin cytoskeleton in this process [2–4]. Although many of the cytoskeletal regulators are conserved between cell types, their precise interactions, effectors, and functions may differ. However, little is known about the role of the DC cytoskeleton in active regulation of IS formation, although there is some evidence for the involvement of cytoskeletal remodeling and the activity of Rho family GTPases Rac1 and Rac2 [5–7]. The DC actin cytoskeleton has been shown to play a role in CD8 T cell activation [8], and most recently, investigation into DC actin’s effects on transmembrane protein mobility has shown that it promotes T cell adhesion [9].

The IS is thought to persist for several hours [10], and there is strong evidence for the involvement of actin and integrins [11]. Despite this apparent stability, the IS is a highly dynamic structure. The macrostructural morphology of the IS can undergo several changes, including T cell pseudopodial extensions deep into the APC [12]. Furthermore, observations of T cell activation in lymph nodes suggest that T cells make multiple contacts with APCs and are thus able to break and reassemble the IS structure several times [13, 14]. The dynamic nature of the IS is also crucial for organization on a much smaller scale, including the movement of transmembrane proteins, such as TCR/peptide MHC, costimulatory molecules, and integrins [15, 16]. On the T cell side, the interface is organized in concentric rings, with TCR in the center (central region of the SMAC), surrounded by a ring of the integrin LFA-1 in the pSMAC. LFA-1 has been implicated in synaptic organization [17–19]. TCR signaling is initiated in peripheral microclusters, whose formation and signaling capacity are highly dependent on actin polymerization [20]. There is also evidence for T cell polarization toward the contact interface, including cell surface markers [7], actin [21], the MTOC [22, 23], and early signaling molecules, such as Lck, ZAP70, and linker for activation of T cells [24–26]. Although the role and precise mechanism of MTOC translocation are unclear, coupling to a functional actin cytoskeleton appears to be essential [27–29].

As actin has been shown to play a key role in IS organization [2, 30–32], it is predicted that actin’s capacity to form stable yet dynamic networks underlies the long-lasting and flexible synaptic structure. The main determinants of cortical actin’s structural integrity and mechanics are the length of actin filaments, the cross-linked nature, and the density of the actin network [33–35]. Actin regulatory proteins control the delicate balance between a dynamic and stable network. One of the best-characterized actin regulators is WASp, expressed exclusively in hematopoietic cells. In T cells, WASp has been proposed to play a role in lipid raft regulation [36], actin-mediated synapse organization downstream of CD2 [37], and induction of symmetry by opposing the effects of protein kinase C θ [38]. More recently, work in T cells has shown that WASp is required for the formation of actin foci associated with T cell signaling [39].

Disturbing the function of WASp in DCs would be expected to result in abnormal cellular actin dynamics and the formation of less stable and poorly organized IS. In this study, we investigate the composition of the DC actin cytoskeleton at the IS and its contribution to IS formation in antigen-specific conjugates using several novel techniques.

## MATERIALS AND METHODS

### BMDC, T cell isolation, and DC:T cell cocultures

BMDCs from C57BL/6 (WT), WASKO, or phosphorylation-null WASp knockin (Y293F [40]) mice were generated, matured with LPS, and pulsed with OVA, as described previously [7]. CD4^+^ T cells were isolated from spleen and lymph nodes of OT-II mice (OVA_323–339_ peptide/I-A^b^-specific CD4 T cells; Charles River Laboratories, Wilmington, MA, USA) by use of a negative selection magnetic bead isolation kit (Miltenyi Biotec, Bergisch Gladbach, Germany). All animals were handled in strict accordance with good animal practice, as defined by UK Home Office Animal Welfare Legislation, Animals (Scientific Procedures) Act 1986, and all animal work was approved by the Institutional Research Ethics Committee (Institute of Child Health, University College London, United Kingdom) and performed under Project License Number 70/7024. For confocal microscopy, DCs and T cells were mixed in a 1:5 ratio and centrifuged gently at 30 g for 5 min to enhance contact formation. The suspension was incubated at 37°C for an indicated time; loose pellets were resuspended gently and seeded on poly-l-lysine-coated glass coverslips for staining and analysis.

### Immunofluorescence staining

Cells were allowed to adhere onto poly-l-lysine-coated coverslips and fixed in 4% PFA for 30 min. Cell membranes were permeabilized in 0.1% Triton for 5 min. To block FcRs, coverslips were incubated with PBS containing 5% serum from the secondary antibody host (donkey, goat, rat, or rabbit) for 20 min. After washing off blocking agents, the primary antibody was added at 1/50–1/100 dilution in PBS–5% serum for 1 h at room temperature. This was washed off with 3× 5-min washes in PBS, and secondary antibody was added at a dilution of 1/100–1/200. Other staining agents, such as DAPI (DNA) and fluorescently tagged phalloidin (F-actin), were also added at this stage. After 45 min, the washing was repeated, and coverslips were mounted using Aqua-Poly/Mount (Polysciences, Warrington, PA, USA). For analysis of podosomes, coverslips were coated with fibronectin (10 µg/ml) for 1 h at 37°C or overnight at 4°C. Coverslips were washed in PBS, and primary DCs were seeded for up to 2 h at 37°C. Once adhered, cells were fixed and stained following the protocol above. The following primary or directly conjugated antibodies were used: anti-capping protein (AB6016; EMD Millipore, Billerica, MA, USA), anti-CD11a (clone I21/7; Leinco Technologies, St. Louis, MO, USA), anti-CD4-allophycocyanin (clone GK1.5; eBioscience, San Diego, CA, USA), anti-CD54-biotin (clone YN1/1.7.4; BioLegend, San Diego, CA, USA), anti-Ea52-68 peptide on I-A^b^ (eBioY-Ae; eBioscience), anti-I-A^b^-biotin (KH74; BD PharMingen, San Diego, CA, USA), anti-TCR (H57-597; Caltag Laboratories, Carlsbad, CA, USA), and anti-vinculin (V4505; Sigma-Aldrich).

### Serial block-face scanning electron microscopy

DC:T cell conjugates were fixed using a combination of 2% PFA and 2% gluteraldehyde for 1 h at room temperature. Samples were washed and embedded in 2 mm cubes of low-gelling temperature agarose (A4018; Sigma-Aldrich). The resulting agar blocks were washed (each wash referred to here comprises 5× 3-min washes) in cold 0.15 M cacodylate buffer with 2 mM calcium chloride. These were then incubated for 1 h on ice in 0.3 M cacodylate buffer with 4 mM calcium chloride combined with an equal volume of 2% aqueous osmium tetroxide and 3% potassium ferrocyanide. After the heavy-metal incubation, samples were washed in dH_2_O and placed in filtered thiocarbohydrazide solution for 20 min. Samples were washed and placed in 2% osmium tetroxide in dH_2_O for 30 min for a second osmium stain. After washing, samples were incubated in 1% uranyl acetate at 4°C overnight. On the following day, Walton’s [41] en bloc lead aspartate staining was performed for 30 min, as described previously. Finally, samples were washed and dehydrated in an ethanol series and infiltrated with Durcupan ACM resin (D036; TAAB Laboratories Equipment, Berks, United Kingdom). These were embedded in a fresh, thin layer of resin and cured overnight at 60°C. Trimmed resin blocks were mounted onto cryopins using cyanoacrylate glue and sputter coated with ∼5 nm gold palladium using a Cressington 108R unit. Imaging was performed using a Gatan 3View low-voltage BSED under variable pressure at 15 Pa and 4 kV. Image stacks comprising 300–1000 aligned BSED images at 100 nm intervals were acquired at a magnification of 50,000× and pixel dwell time of 10 μs.

### FRAP

DC:T cell conjugate suspensions were seeded in glass-bottom imaging dishes. Successful conjugates were chosen for FRAP on the basis of sufficient fluorescent construct expression. Images were acquired every second with a digital zoom of 27× (5 × 5 μm area). Experiments were performed using a 1.4 NA 63× oil-immersion objective on a laser-scanning confocal microscope (Inverted Zeiss LSM 710). Prebleach images were acquired for 5 s, followed by bleaching of a 1 × 1 μm circular ROI using 5 iterations of 100% laser power with the 488 laser (25 mW). Fluorescence recovery was monitored for 2 min at 1 frame/s. In all cases, loss of fluorescence as a result of imaging was significantly smaller than the rates of fluorescent recovery. FRAP data analysis and imaging-induced photobleaching were performed as detailed in Fritzsche and Charras [42].

### Lipid bilayers

Glass-supported planar lipid bilayers were prepared as described previously [43]. In brief, liposome stocks containing 1,2-dioleoyl-*sn*Glycero-3-phosphoethanolamine-*N*-(cap biotinyl) and 1,2-dihexanoyl-*sn*-glycero-3-phosphocholine (both from Avanti Polar Lipids, Alabaster, AL, USA) at a molar ratio of 1:100 were added to clean glass. Following washing, the bilayers were incubated with 5 μg/ml streptavidin for 15 min, washed in PBS, and incubated with biotinylated antigen (anti-MHC II-Cy5 and/or anti-ICAM-1 antibodies) at 1 μg/ml for 30 min. Chambers were washed in HBSS containing 1% BSA, allowing complete buffer exchange, and incubated at 37°C before addition of DCs.

### Online Supplemental materials

Supplemental materials to this manuscript contain extended Methods, Supplemental figures, and videos of podosome live imaging. Supplemental figures include DC maturation status and antigen-presentation assays and FRAP recovery and fluorescence loss curves.

## RESULTS

### DC WASp is required for correct T cell IS formation

**Figure 1.**
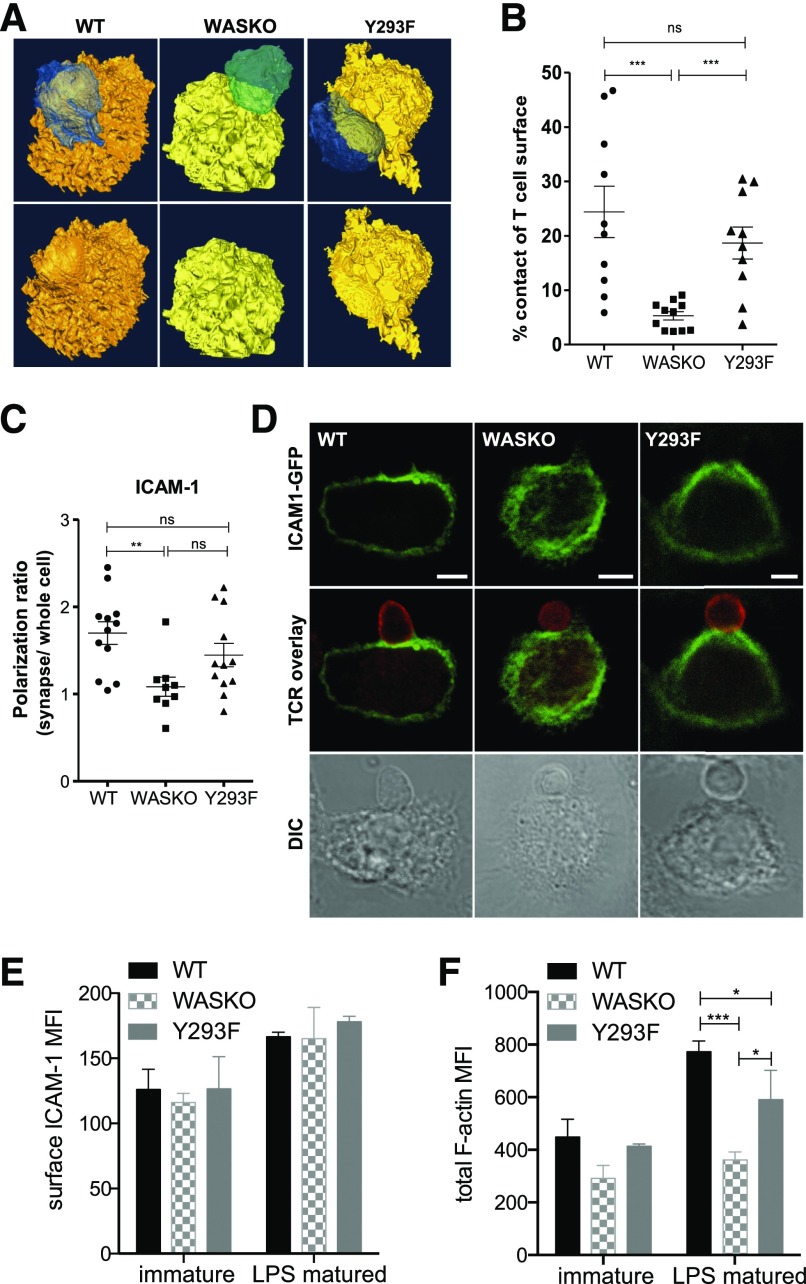
The WASp-dependent DC actin cytoskeleton contributes to correct organization of adhesion molecules and formation of an extensive cell:cell contact. (A) WT, WASKO, and Y293F DCs (yellow/orange), pulsed with OVA, were cocultured with OT-II T cells (blue/green). After 1 h, conjugates were fixed, processed, and imaged using Gatan 3View. Isosurface reconstructions were created in Amira. (Lower) Conjugates with T cell removed to visualize the contact interface. (B) Quantification of DC:T cell contact surface area as a percentage of T cell surface. A minimum of 10 conjugates was analyzed per group. Unpaired *t* test was used to test significance among DC types; ***P(WT + WASKO) = 0.0005, *P*(WASKO + Y293F) = 0.0002; ns *P*(WT + Y293F) = 0.3153. (C and D) DCs expressing ICAM-1-GFP (green) were cocultured with T cells (red; anti-TCR immunostain) for 45 min and fixed. Images represent a slice cutting through the synapse. Polarization ratios of ICAM-1 on the DC side were calculated by measuring fluorescence intensity at the synapse normalized to whole cell. Original scale bars, 5 μm. Unpaired *t* test was used to test significance among DC types; **P(WT + WASKO) = 0.0026; ns, *P*(WT + Y293F) = 0.1875, *P*(WASKO + Y293F) = 0.0610. DIC, Differential interference contrast. (E) Total surface ICAM-1 was measured by flow cytometry in immature and LPS-matured BMDCs, gated on CD11c-positive cells. Means and sd are shown from 3 independent experiments. MFI, Mean fluorescence intensity. (F) Total polymerized actin was measured using phalloidin in permeabilized, immature and LPS-matured BMDCs. Staining was performed in mixed population samples using CFSE labeling. Bars represent means and sd from 3 experiments. ***P(WT + WASKO) = 0.0010, *P(WT + Y293F) = 0.0472, *P(WASKO + Y293F) = 0.0407.

Previously, we have shown reduced migration of WASKO DC and reduced stability and organization of WASKO DC:T cell IS using confocal microscopy [7]. Here, we used serial block-face scanning electron microscopy to examine the DC:T cell synapse in high resolution (**Fig. 1A**). BMDCs from WT, WASKO, or a phosphorylation-null WASp knockin mutant (Y293F) [40], presenting OVA, were used for antigen-specific conjugates with CD4^+^ cells from OT-II mice. With the use of the Gatan 3View technology [44], samples were sectioned with an ultramicrotome, and each consecutive block surface was imaged. WT DCs induced T cell spreading, thus increasing the contact surface area (Fig. 1B and Supplemental Fig. 1A). These conjugates showed very close apposition of the T cell and DC membranes across the whole IS site. WT DCs also underwent cytoskeletal rearrangement to allow the T cell to settle in a "pocket" on the DC surface (Fig. 1A, lower). In contrast, WASKO DCs induced minimal T cell spreading and formed a significantly smaller, less intimate contact interface. Y293F DCs induced an apparently normal contact, suggesting that the presence of WASp, although not its phosphorylation, is sufficient for this process (Fig. 1A and B).

We next wanted to investigate differences in surface molecule organization responsible for the distinct cell:cell contact morphology. We have shown previously a reduction in T cell LFA-1 polarization toward WASKO DC compared with WT [7]. As WASp deficiency was restricted to DCs, here, we investigated polarization of the LFA-1 ligand ICAM-1 on the DC surface, which has previously been shown to direct specific synaptic organization in the cytotoxic IS [45]. WT DCs polarized ICAM-1 toward the T cell contact, but the polarization ratio was reduced significantly in WASKO DC conjugates (Fig. 1C and D) [7]. Y293F DCs showed intermediate polarization, implicating a role for WASp independent of phosphorylation. WASKO DCs showed a significant reduction in ICAM-1 polarization at 15, 30, and 60 min time points (data not shown), suggesting that WASKO DCs were not simply slower but were altogether unable to stabilize a polarized nature. The differences detected were not a result of surface ICAM-1 expression, as all 3 strains showed similar total surface ICAM-1 levels (Fig. 1E).

Differences in IS formation among WT, WASKO, and Y293F DCs were not related to maturation status, as cells displayed similar levels of surface MHC II, CD80, and CD86 (Supplemental Fig. 1B). Furthermore, uptake, processing, and presentation of soluble antigen were found to be normal in WASKO DCs (Supplemental Fig. 1C), as described previously [46]. As expected, the total level of polymerized actin was lower in WASKO DCs compared with WT (Fig. 1F).

Collectively, these data show that reduced ICAM-1 polarization in WASKO DCs is associated with a decreased cell:cell contact area, which is not exclusively dependent on WASp phosphorylation.

### WASp-dependent regulation of the actin cytoskeleton plays a crucial role in IS organization and function

**Figure 2.**
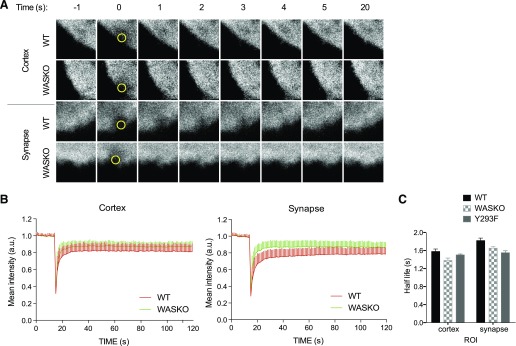
The dynamics of individual actin networks determine synapse stability. (A) Actin-mCherry-expressing WT, WASKO, and Y293F DCs were cocultured with T cells and imaged on a Zeiss LSM 710 microscope. FRAP was performed with 5 iterations of 100% laser power of the 488 laser. ROIs were chosen in the cortex or synapse areas of individual DCs. Actin recovery is followed as the mean mCherry intensity over time within the ROI. Representative images are shown for WT and WASKO DCs bleached at the cortex and synapse. Imaging area = 5 × 5 μm; ROI (yellow circles) = 1 μm^2^. (B) Curves present the means and sd of actin-mCherry fluorescence from a minimum of 45 curves per sample from 3 experiments. For clarity, only WT and WASKO curves are shown. (C) Fluorescent recovery half-life measured at the steady-state cortex and synapse from a minimum of 45 curves from 3 experiments.

To investigate the dynamics of the actin cytoskeleton in WT, WASKO, and Y293F DCs, we used FRAP. Actin-mCherry-expressing DCs were bleached at 2 different regions: cortex (outside of the IS) and IS. Representative images of these are shown in **Fig. 2A**, and their fluorescence recovery curves are plotted in Fig. 2B.

WT DCs showed slower recovery at the IS compared with WASKO, whereas no difference was seen at the cortex. This suggests that the actin cytoskeleton at the IS is more stable compared with that in the steady-state cortex. The reduced stability in the WASKO IS highlights the involvement of actin in this structure and implicates an additional regulatory mechanism at the IS compared with the rest of the cell cortex.

Average half-life values are shown in **Table 1** (column τ_1/2_) and plotted in Fig. 2C. Although there is a trend for reduced half-life in WASKO and Y293F DCs, statistical comparison in this analysis is limited by the frame rate (1 s). Following fluorescence, recovery at 8 frames/s revealed a significantly shorter half-life in WASKO and Y293F DC synapses. This implies an increased turnover and thus, decreased stability of the actin network in these cells.

**TABLE 1.** Parameters of the second-order exponential fit of FRAP recovery curves for different samples

Multiexponent fit		ω_d,1_ (s^−1^)	*P*	*f*_1_	ω_d,2_ (s^−1^)	*P*	*f*_2_	τ_1/2_ (s)	*n*
Cortex	WT	1.28 ± 0.1	–	0.78 ± 0.08	0.21 ± 0.02	–	0.22 ± 0.02	1.65	46
	WASKO	1.28 ± 0.1	0.99	0.78 ± 0.08	0.23 ± 0.02	0.98	0.20 ± 0.02	1.48	50
	Y293F	1.42 ± 0.1	<0.01	0.80 ± 0.08	0.23 ± 0.02	0.99	0.22 ± 0.04	1.52	45
Synapse	WT	0.81 ± 0.2	<0.01	0.81 ± 0.08	0.22 ± 0.02	0.96	0.19 ± 0.02	1.85	58
	WASKO	1.01 ± 0.1	<0.01	0.81 ± 0.08	0.22 ± 0.02	0.98	0.19 ± 0.02	1.66	52
	Y293F	1.20 ± 0.1	<0.01	0.81 ± 0.08	0.22 ± 0.04	0.98	0.19 ± 0.02	1.53	45

Statistical significance of rates of recovery, ω_d,1_ and ω_d,2_, is given in terms of corresponding *P* values. The protein abundances (proportion of each network), *f_i_*, are shown in columns next to each exponent. τ_1/2_, Average half-life value.

In more detailed analysis, the fluorescent recovery of actin can be represented as the sum of a number of exponential functions, where each represents a different process contributing to recovery [47] (Supplemental Methods). At an acquisition rate at 1 frame/s, the fluorescence recovery measured results solely from reactive recovery, rather than diffusion. To determine how many molecular processes contributed to actin turnover, fluorescence recovery was fitted with a combination of exponential functions (detailed in Supplemental Methods). For the DC cortex and synapse, fitting parameters and their *P* values showed a second-order exponential fit, confirming the presence of 2 distinct actin networks (**Table 2** and Supplemental Fig. 2); 1 describes a short-filament, fast-recovery network, and the other corresponds to a long-filament, slow-recovery network. Thus, by separating the components of the recovery curve, the rates and proportions of the separate actin networks contributing to recovery can be calculated (Fig. 2).

**TABLE 2.** Fitting parameters R^2^*_n_*_,order_ and Χ^2^*_n_*_,order_ for first (1st)-, second (2nd)-, and third (3rd)-order exponential fit and corresponding *P* values

Multiexponent fit		*R*^2^_1st_	Χ^2^_1st_	*R*^2^_2nd_	Χ^2^_2nd_	*R*^2^_3rd_	Χ^2^_3rd_	*n*
Cortex	WT	0.90	0.1	0.99	0.001	0.99	0.001	46
	WASKO	0.92	0.1	0.99	0.002	0.99	0.002	50
	Y293F	0.89	0.1	0.99	0.004	0.99	0.003	45
Synapse	WT	0.89	0.1	0.99	0.001	0.99	0.001	58
	WASKO	0.94	0.1	0.99	0.004	0.99	0.004	52
	Y293F	0.90	0.1	0.99	0.003	0.98	0.003	45

At the steady-state cortex, the ω and proportion of each actin network were the same in WT and WASKO DCs. At the IS with a T cell, however, recovery of the fast actin network in WT DCs slowed down (0.81 ± 0.2; Fig. 2 and Table 1) compared with the steady-state cortex, suggesting increased stability in the structure, mainly related to short, branched actin. The recovery rate of this network in WASKO DCs increased (1.01 ± 0.1), showing a perturbation in the stability of branched actin. This confirms that the short time-scale recovery is Arp2/3 mediated and points to an IS-specific role for WASp in actin network organization or stabilization. In Y293F DCs, the rates of recovery appeared faster, suggesting an even higher turnover of branched actin filaments. At this stage, it is unclear whether this represents a compensatory feedback loop through an independent WASp-activating mechanism.

The protein abundance of each network component is given in Table 1 (columns *f*_1_ and *f*_2_). The proportion of fast-recovery filaments increased at the synapse compared with the steady-state cortex, suggesting an increase in short, branched actin upon IS formation.

With the use of this novel approach for investigating IS actin dynamics, we have been able to separate the effects of WASp deficiency on the short and long filament actin networks and demonstrate the crucial role of WASp and Arp2/3-mediated F-actin in DC actin cytoskeleton organization at the IS.

### DCs interacting with supported planar bilayers create IS-like structures whose organization is dependent on WASp

**Figure 3.**
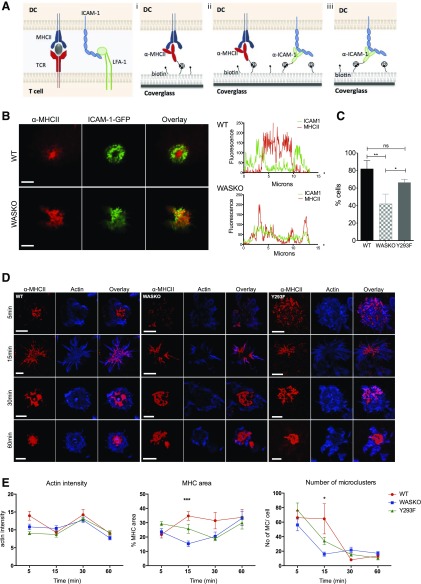
The development of a novel imaging system of the DC synapse. (A) Three different lipid bilayer compositions were designed to mimic a DC:T cell synapse. A biotinylated α-MHC II-Cy5-conjugated antibody was incorporated to replace TCR interaction (Fig. 2). Alternatively, both α-MHC II-Cy5 and α-ICAM-1 antibodies were added to replicate adhesion forces (A and E). α-ICAM-1 alone was used in D. (B) ICAM-1-GFP-expressing DCs interacting with an α-MHC II bilayer were fixed after 20 min and imaged. Original scale bars, 5 μm. Fluorescence intensity of ICAM-1-GFP and MHC II-Cy5 is plotted along the cell diameter, showing differential distribution in WT and WASKO DC. (C) Number of cells exhibiting radial symmetry, as a percentage of cells interacting with the α-MHC II bilayer. A minimum of 30 cells per strain was analyzed. Cells with radial symmetry were defined as having at least 3 different diameter cross-sections showing plots similar to WT DC in B. *P = 0.0232; **P = 0.0092; ns, *P* = 0.0572. (D) WT, WASKO, and Y293F DCs interacting with the α-MHC II-Cy5 (red) bilayer were fixed and stained with phalloidin (blue). Original scale bars, 5 μm. (E) Three parameters were measured by use of ImageJ “Measure” and “Analyze particles” functions in cells interacting with an α-MHC II-Cy5 bilayer: average actin intensity across the contact; MHC II area as a percentage of the total contact area (actin); and number of peripheral microclusters (MC) per cell (size < 600 nm^2^). Means and sem are shown for a minimum of 25 cells per condition analyzed in 2 experiments. ***P(WT + WASKO) < 0.0001, *P*(WASKO + Y293F) = 0.0061, *P*(WT + Y293F) = 0.0664; *P(WT + WASKO) = 0.0113, *P*(WASKO + Y293F) = 0.0084, *P*(WT + Y293F) = 0.0769.

To investigate IS organization with high spatial resolution in the plane of the interface, we developed a novel lipid bilayer system containing anti-MHC II, anti-ICAM-1, or both biotinylated antibodies linked to a biotinylated bilayer using a streptavidin bridge (**Fig. 3A**). On bilayers containing anti-MHC II only (Fig. 3Ai), WT DCs expressing ICAM-1-GFP form a centralized MHC II cluster, surrounded by a peripheral ring of ICAM-1 clusters, mirroring the organization of a T cell IS (Fig. 3B). WASKO DCs were unable to form these distinct molecular rings, as seen in intensity plots across the cell diameter. The proportion of cells forming this radially symmetric organization is quantified in Fig. 3C, showing a significant reduction in the WASKO strain. Y293F DCs presented an intermediate phenotype, with a significantly higher proportion of cells forming symmetric synapse compared with WASKO DC.

Time course analysis showed that MHC II centralization by WASKO DCs was significantly slower and less organized compared with WT (Fig. 3D). Y293F DCs consistently formed intermediate synapses, in terms of symmetry and roundness, suggesting some compensation independent of WASp phosphorylation. The spiked cell morphology at 15 min reflects a cell contraction phase, which in DCs, appeared to last for several minutes and is eventually replaced by a rounded contact. This contraction mechanism is intact in WASKO and Y293F cells and may be regulated by a longer filament formin-mediated actin cytoskeleton, which exhibits normal dynamics at the synapse in the absence of functional WASp (Fig. 2). Figure 3E shows MHC II area as a percentage of total cell area in the proximity of the bilayer, highlighting the difference in MHC II clustering among the WT, WASKO, and Y293F DCs, in particular, at 15 min. The increased proportion in the MHC II area at this time point appears to reflect a sustained production of peripheral microclusters rather than centralized clustering. The number of microclusters was reduced significantly in WASKO and Y293F DCs, correlating with a reduction in the MHC II area (Fig. 3E, right). This confirmed that WASp and the underlying DC actin network play a role in surface MHC II organization at the contact interface.

### WASp- and ICAM-1-dependent podosomes stabilize the IS structure

**Figure 4.**
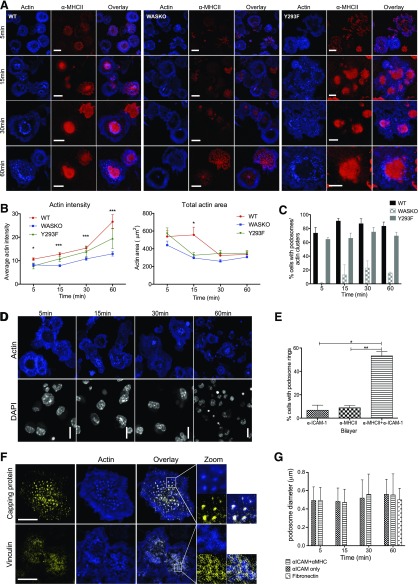
Novel actin organization at the DC synapse. (A) WT, WASKO, and Y293F DCs interacting with an α-MHC II-Cy5 (red) and α-ICAM-1 bilayer were fixed and stained with phalloidin (blue). Original scale bars, 5 μm. (B) The polymerized actin was stained with phalloidin, and fluorescent intensity at the contact site as well as total actin area was quantified at the 4 time points. A minimum of 100 cells was analyzed in 4 experiments; means and sem are plotted. For actin intensity: *5 min: *P*(WT + WASKO) = 0.0113, *P*(WT + Y293F) = 0.0428; ***15 min: *P*(WT + WASKO) = 0.0005, *P*(WASKO + Y293F) = 0.0256; ***30 min: *P*(WT + WASKO) = 0.0007; ***60 min: *P*(WT + WASKO) = 0.0009. For total actin area: *15 min: *P*(WT + WASKO) = 0.0346. (C) Percentage of WT, WASKO, and Y293F DCs forming podosomes on an α-MHC II and α-ICAM-1 bilayer. A minimum of 400 cells was analyzed at each time point. “Actin clusters” are irregular, high-intensity actin structures, similar to those in WASKO cells at 30 and 60 min (A). (D) WT DC contacting an α-ICAM-1-only bilayer. Position of cells is depicted by DAPI staining (white). Phalloidin staining (blue) shows podosome rosettes. (E) The proportion of WT cells forming rings of podosomes in the 3 bilayer conditions is quantified. Means and sem from 3 experiments are shown; a minimum of 300 cells was analyzed. *P = 0.0241, **P = 0.0073 (F) WT DC contacting an α-MHC II-Cy5 and α-ICAM-1 bilayer, showing actin-rich podosomes (blue) and immunofluorescent staining (yellow): capping protein (F-actin capping protein, α subunit; upper), vinculin (lower). Colocalization of F-actin capping protein and actin produces a white overlay; 36 WT DCs were analyzed to calculate colocalization. Pearson correlation coefficient = 0.442 ± 0.14; Mander’s overlap coefficient = 0.777 ± 0.04. Original scale bars, 5 μm. A 3× zoom is shown to the right. (G) DCs were seeded on 2 different bilayers and on fibronectin (50 μg/ml) and fixed at set intervals. Diameter of the podosome actin cores was measured in ImageJ; >100 podosomes were measured for each condition. Synapse podosomes did not change significantly over time and showed a similar size to those formed on the ventral side of cells adhering to fibronectin.

To mimic the DC:T cell contact more completely, we added anti-ICAM-1 to the lipid bilayer before allowing DCs to interact (Fig. 3Aii). Once again, WASKO DCs showed slower and less symmetric MHC II organization (**Fig. 4A**). Engagement of this integrin ligand induced more pronounced cell spreading in WT DCs, resulting in a larger total area compared with WASKO DCs (Fig. 4B, right), concurrent with poor ICAM-1 organization by WASKO DCs. Surprisingly, the WT DCs formed actin-rich, podosome-like structures at all time points analyzed, which correlated with an increase of phalloidin fluorescence intensity at the contact (Fig. 4B). The proportion of cells forming podosome-like structures is quantified in Fig. 4C, where actin clusters represent larger, irregular actin clusters, such as those seen in WASKO DC at 15, 30, and 60 min.

Over time, podosomes assembled into a distinct ring surrounding the central MHC II cluster, and crucially, this organization was dependent on engagement of ICAM-1 and MHC II (Fig. 4E). Contact with anti-ICAM-1-only bilayers induced podosome-like structures that formed clusters or rosettes rather than rings (Fig. 4D). In the absence of ICAM-1 ligation, podosomes-like structures did not form at any time point (Fig. 3D). These podosome-like structures were completely absent in WASKO DCs (Fig. 4A). To characterize these actin-rich structures further, we used immunostaining for vinculin (Fig. 4F), which was present in rings surrounding the individual actin structures, similar to canonical podosomes described elsewhere [48, 49], suggesting that these actin-rich structures represent true podosome cores. Staining for F-actin capping protein, α subunit 1 localized to the actin-rich podosome cores (Mander’s overlap coefficient, 0.777 ± 0.04), highlighting that Arp2/3 nucleation, polymerization, and filament capping are important for these podosome structures. Furthermore, the podosome diameter was similar to that of classic podosomes formed on fibronectin (Fig. 4G), as reviewed in ref. [49]. Live imaging of actin-mCherry-expressing DCs illustrated the dynamic nature of individual podosomes at the IS in contact with a bilayer; WASKO DC interacting with the same bilayer and a migrating DC are shown for comparison (Supplemental Movies 1–3).

### Functional consequences of abnormal DC IS

**Figure 5.**
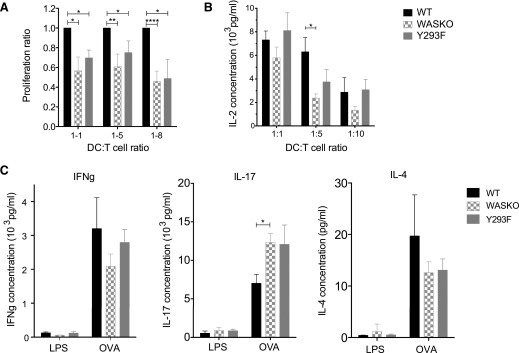
Abnormal synapse formation has functional consequences for T cell activation. (A) OVA-pulsed DCs were cocultured with CFSE-labeled T cells at 3 different DC:T cell ratios (1:1, 1:5, 1:8) for 48 h. Proliferation was measured by following CFSE dilution in the CD4^+^ population. Proliferation ratios were calculated from 5 independent experiments by determining the proportion of proliferated cells (above LPS-only control) and normalizing this to the WT value for each respective experiment. *P = 0.01–0.05; **P = 0.0095; ****P < 0.0001. (B) IL-2 secretion in supernatants from DC:T cell cocultures was measured using the ELISA kit (R&D Systems, Minneapolis, MN, USA) in 3 individual experiments in triplicate. A paired *t* test was used to calculate significance between WT and WASKO cocultures. *P = 0.0460. (C) Supernatants were collected from 1:1 DC:T cell suspensions, 48 h after coculture and tested for cytokine secretion by ELISA. Samples were tested in triplicates, and graphs represent the means and sem of 4 separate cocultures. A paired *t* test was used to calculate significance. *P = 0.0245 (IL-17).

Several studies have suggested that varying the strength of the T cell contact with DCs, either through antigen dose or antigen affinity for TCR, can alter Th cell differentiation [50–52]. Fate induction may also depend on successful activation of costimulatory receptors, such as LFA-1, CD28, or CTLA-4 [52–57]. Therefore, abnormal spatial or temporal organization of these is likely to disturb downstream signaling. The malformed IS in the absence of WASp was shown to be insufficient for full T cell activation. WASKO DCs induced less T cell proliferation and IL-2 secretion (**Fig. 5A** and **B**). The differences were particularly striking at lower DC:T cell ratios, suggesting that the DC actin cytoskeleton is especially important in conditions with reduced levels of DC-induced stimulation. Some partial recovery was seen with Y293F DCs, reflecting the intermediate phenotype in synapse organization. We also found that WASKO DCs induced slightly decreased IFN-γ and IL-4 secretion but significantly increased IL-17 secretion in vitro in DC:T cell cocultures compared with WT DCs (Fig. 5C). This highlights potentially important, functional downstream consequences of a disorganized DC cytoskeletal rearrangement.

## DISCUSSION

How cells form stable interactions for effective intercellular signaling is an important question in cell biology and the field of immunology in particular. In the context of DC–T cell IS formation, whereas the role of the T cell cytoskeleton has been well described, few studies have explored the importance of the DC as an active rather than passive participant [5, 6, 58]. To our knowledge, our study is the first to visualize the DC side of the IS at this resolution.

Actin is important for cell integrity, transmembrane protein clustering, membrane organization, and mechanosensing. Thus, dysregulated actin polymerization or a reduced total F-actin network may make DCs less responsive to external physical cues and less able to form precise structures by transporting or stabilizing transmembrane proteins. This appears to be the case for ICAM-1, as its polarization toward the IS is defective in WASKO DCs. The expected polarization of the MTOC and LFA-1 in WT-conjugated T cells was also markedly diminished [7], indicating an important driving role for the DC in molecular organization of the adjacent T cell synaptic interface. Indeed, recently, Comrie et al. [9] showed that decreased ICAM-1 mobility in mature DCs results in high-affinity LFA-1 on the T cell side.

A defect in integrin polarization and therefore, reduced IS integrin density is likely to result in decreased adhesion, which would explain the lower number of stable conjugates formed by WASKO DCs (data not shown) [7]. This is supported by atomic force microscopy, showing that the blocking of LFA-1 function abolishes interaction forces in T cell–APC conjugates [59]. Reduced integrin binding at the IS also accounts for the reduced contact interface area seen by serial block-face scanning electron microscopy by minimizing or eliminating the pSMAC in WASKO DC conjugates.

The dysregulated integrin organization and adhesion in WASKO DCs may contribute to their inability to induce T cell MTOC translocation, as we have shown previously [7]. Whereas early studies suggested the process was dependent on TCR signaling [24–26], more recently, Yi et al. [60] demonstrated a requirement for LFA-1 for MTOC polarization. Further evidence is provided by studies of l-plastin, the actin-cross-linking molecule whose phosphorylation has been linked to LFA-1 localization and activation [61, 62]. The blocking of l-plastin function results in abnormal IS formation, cytokine secretion, and MTOC docking [63], remarkably similar to results shown in WASKO DCs here.

The marked structural differences at the IS are clearly associated with altered actin dynamics in WASKO DCs. The actin cytoskeleton provides a mechanical platform for IS signaling. Here, we show that actin at the cortex and synapse of DCs comprises 2 separate networks, as demonstrated in other cell types [47]. In the context of FRAP recovery, the short Arp2/3-mediated filaments are dominant on short time scales and the long formin-mediated filaments on long time scales. Consistent with this, perturbations in WASp affect the fast-recovery, Arp2/3-nucleated filaments. In WT DCs, actin recovery was slower at the IS compared with the steady-state cortex, implicating a distinct actin regulatory mechanism during IS formation or maintenance. The absence of WASp has a minimal effect on the cortex but significantly increases the recovery rate of actin in the IS, pointing to a less stable actin network. WASKO DCs also contain less total polymerized actin. Taken together with the increased recovery rate of F-actin, this implies faster turnover and reduced network stability, which are likely to translate into observed defects in macromolecular organization, such as the abrogated recruitment and concentration of MHC II molecules to the contact interface on supported bilayers. This would be the case regardless of whether the role of actin in IS organization involves retrograde flow, size exclusion, or simply anchoring for membrane protein clusters. Interestingly, the Y293F mutant produced fast recovery times and did not recover actin network stability, consistent with a requirement for WASp phosphorylation for full Arp2/3-mediated actin polymerization. This suggests that the partial recovery phenotypes, such as ICAM-1 polarization, contact interface, and T cell activation, result from phosphorylation-independent WASp activation or WASp functions unrelated to actin polymerization.

Podosomes are specialized cell adhesion structures containing an actin core surrounded by a ring of integrins, scaffold, and actin-binding proteins [48, 49]. The role of podosomes in cell adhesion is well described in osteoclasts [64] and migrating cells [65] but has not been implicated in IS structure or function. The existence of invasive pseudopodia from the T cell deep into the DC appears to be an important early phenomenon during formation of an IS, although their function remains unknown [12]. How these relate to podosomes at the interface, if at all, is also unclear, although recent evidence suggests that podosomes may have an important role in sensing the stiffness of substrata and modulating cell behavior through mechanotransduction [66]. As it is technically difficult to visualize podosomes in true in vivo settings, further improvements in cell contact imaging will be necessary to confirm the presence of podosomes when contacting a more deformable, intact cellular membrane rather than an artificial bilayer. The function of podosomes at the IS cannot, as yet, be accurately defined. Although podosomes have been implicated in the transfer of extracellular matrix digesting enzymes, in the context of a conjugated cellular interaction, it would seem much more likely that their function is related more to consolidation and regulation of adhesion. Further work could investigate how these structures compare with other processes, such as formation of a sealing ring at the osteoclasts' interface with bone [64, 67].

Data presented in this study highlight the essential role of the DC actin cytoskeleton in immune cell communication. Functional defects resulting from poor DC-mediated IS organization (Fig. 5) highlight an important role for IS stability in T cell fate induction and therefore, may contribute to the pathophysiology of diseases, such as the WAS, where this is intrinsically compromised. The role of specific actin structures, such as podosomes, may well be important, particularly through regulated adhesion, but need to be characterized in detail in vivo. However, there is a clear driver role for the DC cytoskeleton during IS formation, with implications for T cell priming, activation, or functional fate determination.

## AUTHORSHIP

D.M., A.J.T., G.C., and G.B. designed the experiments. D.M., M.F., C.R.N., H.A., P.M.G.M., and M.P.B. performed experiments. D.M., M.F., and H.A. analyzed data. D.M., A.J.T., and P.T. prepared the manuscript. The authors declare no conflict of interest.

## Supplementary Material

Supplemental Data

## Abbreviations

ω: rate of recovery
Arp2/3: actin-related protein-2/3
BMDC: bone marrow-derived dendritic cell
BSED: backscatter electron detector
DC: dendritic cell
F-actin: filamentous (polymerized) actin
FRAP: fluorescence recovery after photobleaching
IS: immunological synapse
MHC II: MHC class II
MTOC: microtubule-organizing center
PFA: paraformaldehyde
pSMAC: peripheral supramolecular activation cluster
ROI: region of interest
SMAC: supramolecular activation cluster
WAS(p): Wiskott-Aldrich syndrome (protein)
WASKO: Wiskott-Aldrich syndrome (protein) knockout
WT: wild-type
