# Supplementary material for: WASp-dependent actin cytoskeleton stability at the dendritic cell immunological synapse is required for extensive, functional T cell contacts
Source: J Leukoc Biol. 2015 Nov 20;99(5):699–710. doi: 10.1189/jlb.2A0215-050RR (PMC5404712; doi:10.1189/jlb.2A0215-050RR)
Supplement: Supplemental Data [file supp_99_5_699__index.html]

WASp-dependent actin cytoskeleton stability at the dendritic cell immunological synapse is required for extensive, functional T cell contacts — WASp-dependent actin cytoskeleton stability at the dendritic cell immunological synapse is required for extensive, functional T cell contacts — WASp-dependent actin cytoskeleton stability at the dendritic cell immunological synapse is required for extensive, functional T cell contacts — Supplemental Data 

# WASp-dependent actin cytoskeleton stability at the dendritic cell immunological synapse is required for extensive, functional T cell contacts

## Supplemental Data

- Supplemental Data
- Supplemental Data
- Supplemental Data
- Supplemental Data
- Supplemental Data
- Supplemental Data
