# Supplementary material for: WASp-dependent actin cytoskeleton stability at the dendritic cell immunological synapse is required for extensive, functional T cell contacts
Source: J Leukoc Biol. 2015 Nov 20;99(5):699–710. doi: 10.1189/jlb.2A0215-050RR (PMC5404712; doi:10.1189/jlb.2A0215-050RR)
Supplement: Supplemental Data [file supp_jlb.2A0215-050RR_Supplemental_Data.docx]

**Structural analysis of the DC immunological synapse reveals differentially regulated actin cytoskeleton**

Dessislava Malinova^1^, Marco Fritzsche^2^, Carla R Nowosad^3^, Hannah Armer^4^, Peter MG Munro^4^, Michael P Blundell^1^, Guillaume Charras^2^, Pavel Tolar^3^, Gerben Bouma^1^*, Adrian J Thrasher^1,5^*

*^1^Molecular Immunology Unit, UCL Institute of Child Health, 30 Guilford Street, London, WC1N 1EH, UK.*

*^2^London Centre for Nanotechnology and Department of Cell and Developmental Biology, UCL.*

*^3^Division of Immune Cell Biology, MRC National Institute for Medical Research, The Ridgeway, Mill Hill, London NW7 1AA, UK.*

*^4^Imaging Unit, UCL Institute of Ophthalmology, 11-43 Bath Street, London EC1V 9EL, UK.*

*^5^Great Ormond Street Hospital for Children, NHS Foundation Trust, London WCIN3JH, UK.*

**Supplementary materials**

**Supplementary methods**

**Molecular biology**

Second-generation lentiviral packaging vectors were used to produce lentiviruses containing constructs encoding Actin-mCherry and ICAM1-GFP, as described in: Demaison 2002.

For BMDC infection, cells were cultured in GMCSF and on day 5, cells were harvested and replated at 5x10^5^ cells per well (12-well plate) in 500μl of conditioned media. Cells were allowed to settle for 30min at 37°C before addition of lentivirus at an MOI = 5 (multiplicity of infection). After a further 30min incubation, 1.5ml of fresh complete media supplemented with 20ng/ml GMCSF was added. Cells were harvested on day 8 for further experiments.

**FRAP data analysis**

FRAP data analysis was effected as described in [1]. The total fluorescence signal at the actin cortex results from monomers bound within the cortical actin filaments and monomers freely diffusing in the interstice between the cortical actin mesh. Therefore, fluorescence recovery after photobleaching will have contributions from (i) cytosolic diffusion between actin mesh (cytosolic diffusive recovery), (ii) association/dissociation of proteins to the cortex (reactive recovery). Previous reports have measured a diffusion constant of D~30μm^2^.s^-1^ for actin-GFP in the cytoplasm of epithelial cells [2]. In our experimental geometry, diffusion of actin-mCherry should take place with a characteristic time-scale of τ~r^2^/4D~10ms with r=1μm the radius of the bleach zone, several-fold shorter than the characteristic times of the reactions examined in this study (τ≥400ms). Thus, given the acquisition rate used in this study (1s per frame), diffusive recovery was complete by the time we acquired the first post-bleach frame and fluorescence recovery measured was solely due to reactive recovery. To determine how many first order molecular processes contributed to turnover, cortical recovery *F(t)* was fitted with a combination of exponential functions *F_i_* of the form F_i_(t)~1-*exp(-t/τ_d,i_)f_i_F_0_* with F_0_ the initial fluorescence of the bleached region and *i* a molecular process participating to recovery. Each function F*_i_* represents the contribution of the molecular process *i* to the total recovery with *f_i_* the portion of the total protein population undergoing turnover process *i* (Σ_i_f_i_=1), and *τ_d,i_* the characteristic dissociation time of process *i.* The characteristic dissociation time τ_d_ is linked to the half-time reported in most FRAP experiments: t_1/2_=ln(2)τ_d_. The apparent association time *τ_a,i_* can be calculated as *τ_a,i=(_τ_d,i /_* *f_i_)*(F_0_/F)* [1, 2]. If turnover results from association/dissociation of a protein from the cortex, *τ_d_* can also be expressed as an apparent dissociation rate *ω_d_=1/τ_d_*_._ One limitation of this analytical approach is that we can only distinguish molecular processes that occur on sufficiently different time-scales. If several molecular processes occur at similar time-scales, the apparent rate constant measured reflects an average over all of the molecular processes acting at that time-scale. In practise, fluorescence recovery curves *F(t)* were fitted with an increasing number of exponential functions until the three following conditions were met: the goodness of fit estimated through r^2^ no longer increased, the total change in fluorescence associated with process *n* was less than 0.001%, and the sum of squared errors no longer decreased (Table 2). Hence, this approach allows determination of the number of molecular processes *i* that contribute to fluorescence recovery, their characteristic times *τ_d,i_*, as well as the portion *f_i_* of the total protein population that recovers through process *i*. Theoretical derivations and detailed fitting procedures are given in [2].

**Antigen presentation assays**

***Eα-GFP:*** Immature BMDCs were pulsed with varying concentrations (0.01 – 100 μg/ml) of Eα-GFP protein (a gift from P Garside, University of Glasgow) in combination with LPS (as above), on day 7. Eα presentation was detected 24hours later by flow cytometry using an antibody specific for the Eα peptide in context of MHC class II peptide (eBioscience, eBioY-Ae).

***T cell proliferation:*** Isolated CD4+ T cells were stained in a final working concentration of 10μM CFSE. Cells were washed and cocultured with wildtype or WASKO DCs (pulsed with LPS with or without OVA) at ratios of 1:1, 1:5 and 1:8. Three days later, cells were harvested and immunostained for CD4. CFSE content was analysed by flow cytometry, gating on CD4-positive cells only.

***IL-2 and other cytokine production:*** Unlabelled CD4+ T cells were cocultured with DCs for 48hours, as above, at DC: T cell ratios of 1:1, 1:5 and 1:10. Cytokine secretion was measured from 24 and 48hr DC: T cell coculture supernatants using R&D systems Duo Set ELISA kits. 1x TMB solution was added as a substrate for HRP and incubated at room temperature for 15-30minutes. Sulphuric acid stop solution (2N H2SO4) was added and absorbance at 450nm was measured on an Optima FLUOStar plate reader. T cells were activated using Mouse T-Activator CD3/CD28 Dynabeads (Invitrogen, 114.52D), or Concanavalin A (Sigma Aldrich, C5275) as positive controls for T cell activation and proliferation.

**Supplementary figures**

**Supplementary Figure 1**

The reduced capacity of WASKO DCs to induce T cell spreading, polarisation, proliferation and cytokine secretion is not related to maturation state and antigen processing ability.

A) OVA-pulsed WT DCs were cocultured with OT-II T cells for 1 hour and processed for Gatan 3View imaging. Examples of isosurface reconstructions shown here highlight the range of extensive cell: cell contacts induced by WT DCs.

B) Surface MHC-II, CD80 and CD86 were measured by flow cytometry on mature and immature BMDCs. Graphs represent mean and standard deviation from 4 separate cultures.

C) BMDCs were pulsed overnight with LPS and a range of concentrations of Eα peptide. Uptake, processing and presentation are measured by flow cytometry using Yae antibody. Graph represents the average percentage of Yae positive cells from 4 separate experiments.

**Supplementary Figure 2**

Actin-mCherry FRAP recovery curves. Mean and standard deviation for a minimum of 45 curves at each experimental condition are plotted in black. The loss of fluorescence due to imaging is plotted in grey; all curves recover to 100% minus loss of fluorescence due to photobleaching. Second order exponential fit is shown in red.

**Supplementary Movie 1**

Actin-mCherry expressing WT DC interacting with an anti-MHCII and anti-ICAM1 bilayer. Imaging was performed at 37°C over 28min with a 100x objective. Highest intensity actin structures appear in white on the 16-colour LUT. Image size = 81μm^2^.

**Supplementary Movie 2**

Actin-mCherry expressing WT DC seeded on poly-L-lysine and imaged over 7 minutes. Imaging was performed in RPMI without phenol red, in a 37°C heated chamber on Zeiss LSM710 confocal microscope using a 1.4NA 63x oil objective. Image sequence was combined in ImageJ and is shown in 16-colour look up table (LUT). Image size = 41.8μm^2^.

**Supplementary Movie 3**

Actin-mCherry expressing WASKO DC interacting with an anti-MHCII and anti-ICAM1 bilayer. Imaging was performed at 37°C over 28min with a 100x objective. Highest intensity actin structures appear in white on the 16-colour LUT. Image size = 81μm^2^.

1. Fritzsche, M. and G. Charras, *Dissecting protein reaction dynamics in living cells by fluorescence recovery after photobleaching.* Nature Protocols, 2015. **10**(5): p. 660-680.

2. Fritzsche, M., et al., *Analysis of turnover dynamics of the submembranous actin cortex.* Molecular Biology of the Cell, 2013. **24**(6): p. 757-767.
